# Progress in Medicine

**Published:** 1900-02-03

**Authors:** 


### Progress in Medicine.

### FEVERS.

(*Continued from page* 275.)

**Treatment of Typhoid Fever.**—Rousch[9] lays stress on the value of calomel daily for the first week till the characteristic stool is changed. He also gives salicylate of ammonium throughout with all the water, tea, or coffee which the patient cares to take, and cold sponging daily. At first no food is given unless the patient desires it; later on, milk, butter milk, and fruit juice are best to commence with. For tympanites, small doses of calomel and salol, with larger ones of strychnine. J. T. Moore[10] thinks that intestinal antiseptics, such as guaiacol, naphthol, or calomel, chosen to meet individual conditions, should be given, with cold sponging or packs when high temperatures occur. Starch foods should be the last that are permitted during convalescence. Basil Taylor[11] feeds only when hungry, and then only three times a day, with pulped beef, eggs, powdered crackers, beef tea, and soup, as he holds these substances are digested before leaving the stomach. Dyspeptics often do best on white of egg, though unable to digest milk. The colon is irrigated two or three times a day with or without antiseptics, and warm baths are given daily. It is curious to find that at least two American and an Australian writer allow their patients to step from bed into the bath or to the night-stool when no special complications are present. E. F. Wilson, of Ohio,[12] pursues this plan, but otherwise orders complete rest. Since curds of undigested milk are found in the bowels of typhoid patients after death, he either pre-digests it, or in ordinary cases substitutes for it altogether a diet of eggs, meat juice, soup, and farinaceous food, such as arrowroot and fruit juice. He administers at first castor oil or calomel, then salol or some other antiseptic, and if the temperature is very high he orders iced enemata and sponging. For perforation or hæmorrhage morphia is the only remedy which has given him good results. A number of writers recommend baths formed of rubber sheets, which can be slipped under the patient and drawn up to a frame around him so as to avoid the trouble and danger of lifting him in and out of bed, but W. I. Sumner[13] condemns the Brand treatment on the ground of its impracticability for general practitioners and nurses, because he thinks it should be accompanied with proper massage, and on account of its supposed danger for weak hearts. He even takes the generally negatived view that antipyretics are safer in such cases. For dry tongue, meteorism, and ulceration he finds turpentine valuable, and advocates strychnia for heart failure. In spite of his opposition to baths, he employs sponging, and imagines that the type of the disease and its rate of mortality has fallen, but we may refer here to the Massachusetts statistics quoted above.

**Complications.**—Among irregular cases Osler[14] reckons: (*a*) very mild forms, such as occur in most epidemics, where slight fever, headache, and diarrhœa last only a few days. The Widal reaction may fail until the fever is nearly over, but still it is of the greatest use; (*b*) the acute septicæmic form with high fever may end fatally before any diagnostic symptoms appear, even before the Widal reaction can take place, and after death there may be no intestinal lesions; (*c*) cases simulating meningitis, bronchitis, or nephritis may be quite without symptoms of typhoid until the complication has spent itself. Even rigors may occur from many causes and lead us to a wrong diagnosis of malaria, which is an extremely common error in America. Malaria, by the way, yields quickly to quinine, and either shows remissions or a very irregular temperature with early anæmia. The coincidence of the two diseases is very rare, and a hybrid "typho-malarial" is, according to Osler's well-known view, non-existent. Multiple ulcers of the vagina and vulva are reported by G. A. Lartigan[15] in two cases where the general symptoms were well marked. The ulcers were from eight to 12 in number, causing much pain in micturition, and from some of them the typhoid bacillus was isolated.

[9] New York Med. Jour., Sept. 16. [10] Mercks' Archives, Nov. [11] Med. Rec., Sept. 16. [12] Columb. Med. Jour., Oct. [13] Charlotte Med. Jour., Oct. [14] New York Med. Jour., Nov. 4. [15] Lancet, Oct. 7.

